# Anti-inflammatory effect of fluvastatin on polarized macrophages and its dependence on the mevalonate pathway

**DOI:** 10.1038/s41598-025-02418-9

**Published:** 2025-06-02

**Authors:** Barbora Muffova, Sona Kauerova, Karel Paukner, Hana Bartuskova, Rudolf Poledne, Ivana Kralova Lesna

**Affiliations:** 1 Laboratory for Atherosclerosis Research, Centre for Experimental Medicine, Institute for Clinical and Experimental Medicine, Prague, Czech Republic; 2 Department of Physiology, Faculty of Science, Charles University, Prague, Czech Republic; 3 Department of Anesthesia and Intensive Medicine, First Faculty of Medicine, Charles University and University Military Hospital, Prague, Czech Republic

**Keywords:** Fluvastatin, Macrophages, Polarization, Inflammation, Mevalonate pathway, Cell biology, Immunology, Molecular biology

## Abstract

The study focuses on the effects of fluvastatin on immunomarkers of the M1 and M2 macrophages and its direct role in macrophage (M0) polarization. Moreover, it investigates the dependency of immunomodulatory properties of fluvastatin on the mevalonate pathway. Macrophages (M0, M1, M2), differentiated from human blood monocytes, were treated with fluvastatin. Mevalonate and geranylgeranyl pyrophosphate intermediates were introduced to assess the mevalonate pathway dependence. The immunomarkers were evaluated with qPCR, ELISA, Griess assay, and flow cytometry. Fluvastatin significantly reduces the pro-inflammatory gene expression (NFκB, IL-1β, IL-6, iNOS) in M1 while enhancing the anti-inflammatory markers (Arg-1, TGFβ) in M2 macrophages. The production of the TNFα, IL-1β, and IL-6 cytokines is reduced in M1, and IL-10 production increased in M2 macrophages. Fluvastatin decreases the iNOS activity in M1 macrophages. The intermediates reverse the fluvastatin’s effects on anti-inflammatory gene expression by M2 macrophages, cytokine production (by M1 and M2 macrophages), and iNOS activity (by M1 macrophages). Their impact on surface marker expression was somewhat limited. These findings demonstrate that fluvastatin exerts anti-inflammatory effects on polarized macrophages without affecting polarization per se and also highlight the dependency on the mevalonate pathway. This study deepens the understanding of statins’ immunomodulatory mechanisms, suggesting potential applications in treating inflammatory diseases.

## Introduction

Statins, first described by Dr. Akira Endo in the late twentieth century, are potent mevalonate pathway inhibitors crucial in cholesterol synthesis^1^. Endo isolated the first statin, mevastatin, from *Penicillium citrinum*, finding it a competitive inhibitor of HMG-CoA (Hydroxymethylglutaryl-Coenzyme A) reductase, effectively blocking cholesterol production. Statins reduce cholesterol synthesis and increase low-density lipoprotein (LDL) receptor expression, enhancing the removal of LDL cholesterol from the bloodstream and making them essential for managing hypercholesterolemia^2^.

Since the introduction of lovastatin in 1987 as the first statin approved for human therapy, statins have become the most widely used lipid-lowering drugs^3^. Currently, seven statins are in clinical use, with atorvastatin and simvastatin being the most prescribed due to their high efficacy in lowering LDL cholesterol^4^, and pitavastatin and fluvastatin^4^. Although pitavastatin and fluvastatin are less commonly prescribed, they are better tolerated by patients who cannot tolerate other statins^5^. Fluvastatin also shows a lower risk of development of type 2 diabetes compared to other statins^6^.

Besides their lipid-lowering effect, statins influence immunity. The randomized JUPITER (Justification for the Use of Statins in Primary Prevention) trial of healthy individuals with elevated high-sensitivity C-reactive protein without hyperlipidemia found that rosuvastatin significantly reduced the rates of first major cardiovascular events and all-cause mortality compared with placebo^7^. This demonstrates the critical role of low-grade chronic inflammation in cardiovascular disease development while highlighting the protective effects of statins in mitigating this risk. Over the years, research has brought evidence about the anti-inflammatory properties of statins in vitro^8–11^, in animal models^12–14^ and in humans^12,15,16^, with the immunomodulatory properties of macrophages being thoroughly reviewed in Sheridan et al.^17^.

Macrophages are highly dynamic innate immune cells capable of adopting diverse functional phenotypes in response to environmental signals. They are the key mediator of inflammatory and metabolic signals and processes within the atherosclerotic plaque. Macrophages exist across a broad spectrum of phenotypes, where the boundaries between pro- and anti-inflammatory states are less distinct. Their functional plasticity is essential for maintaining immune homeostasis and contributes to disease pathogenesis. Understanding the balance between macrophage phenotypes is crucial for developing therapeutic strategies that enhance immune responses or suppress harmful inflammation. The development, transcriptional regulation, functions, and pathological roles of macrophage subpopulations have been extensively reviewed in prior studies^18,19^.

### AIMS

The rationale of this study was to understand the fluvastatin effect on macrophage inflammation of fully polarized macrophages within an established M1/M2 macrophage polarization in vitro model. The other objective of this study was to investigate whether fluvastatin directly influences macrophage polarization and, if so, to elucidate whether their effects predominantly promote a pro-inflammatory or anti-inflammatory response. Therefore, we decided to add non-polarized M0 macrophages. As it is well known that the classical function of statins depends on the mevalonate pathway, we sought to elucidate if the immunomodulatory properties of fluvastatin also rely on this pathway. To achieve these goals, we examined the impact of statin treatment on various parameters, including the expression of macrophage surface markers, gene expression profiles, cytokine production, and the enzymatic activity of inducible nitric oxide synthase (iNOS) in vitro. Lastly, we investigated the potential reversal of fluvastatin’s effects by supplementing mevalonate and geranylgeranyl pyrophosphate (GGPP).

## Experimental section

### Definition of subjects

Human monocytes were isolated from the buffy coats of healthy blood donors provided by the Department of Transfusion Medicine at Thomayer University Hospital in Prague. The study design was approved by the Institute for Clinical and Experimental Medicine Ethics Committee and Thomayer Hospital, Prague, Czech Republic, and complied with the Declaration of Helsinki. Clinical data were gathered from the participants’ medical records and through interviews focused on lifestyle factors typically assessed before blood donation. All the subjects (n = 13) were male, not older than 35 years, and naïve to statin or hormone therapy.

### Harvesting of human monocytes, differentiation into macrophages, and their subsequent polarization and treatment

Buffy coats were diluted with Phosphate Buffered Saline (PBS; Biosera, Manila, The Philippines) + 2% FBS (fetal bovine serum) in a 1:1 ratio. Mononuclear cells were isolated using Lymphocytes Separation Medium 1077 (PromoCell GmbH, Heidelberg, Germany) in the SepMate™-50 (IVD) tubes for density gradient centrifugation (StemCells Technologies, Vancouver, Canada; centrifuged on 400 g, RT, 10 min [with a break on]). Monocytes were isolated by adhesion to a plastic^20^ for 4 h. Media were aspirated from adhered cells and subsequently washed with cold PBS. The cells were incubated for 6 days in RPMI-1640 (Biosera, Manila, The Philippines) + 10% FBS (Biosera) + 1% P/S (Biosera) + 1% glutamine (Biosera) medium with colony-stimulating factor (M-CSF; Preprotech, Hamburg, Germany; 80 ng/mL)^12^. The medium was changed three times during the 6-day incubation period. After the period, the old media were aspirated, and macrophages were treated with freshly mixed media with agents to polarize them toward pro-inflammatory M1 and anti-inflammatory M2 phenotypes. M1 macrophages were polarized with a combination of lipopolysaccharide (LPS; PreproTech, Hamburg, Germany, 100 ng/mL) and interferon gamma (IFNγ; Gibco, Gaithersburg, MD, USA, 20 ng/mL)^12,21^ whereas the combination for M2 macrophages contained interleukin-4 (IL-4; Preprotech, Hamburg, Germany, 20 ng/mL) and IL-13 (Preprotech, Hamburg, Germany; 20 ng/mL)^12,22^. The third subtype of macrophages was considered non-polarized, as they were not treated with any cytokine (except the previously used M-CSF). After washout of M-CSF, the macrophages were further incubated with or without the addition of fluvastatin (Sigma-Aldrich, 10 µM) and the intermediates of the mevalonate pathway; mevalonic acid (mev; Sigma-Aldrich, 10 µM) or geranylgeranyl pyrophosphate (GGPP; Sigma-Aldrich, 20 µM), with the concentration adopted from Fu et al.^23^. This study added intermediates to investigate the dependency of fluvastatin’s immunomodulatory properties on the mevalonate pathway. The cells were incubated in 12-well plates (with the density seeding 250 × 10^3^ cells/well).

After 48 h of incubation, cells were harvested and centrifuged (300 g, 5 min, RT) and subsequently used for flow cytometry (FC) or quantitative real-time PCR (qRT-PCR). Media were centrifuged (300 g, 5 min, RT) and stored at − 80 °C for further analysis. In total, macrophages were derived from 13 healthy human donors.

### Surface marker expression—flow cytometry (FC)

The cells were harvested as described above. The cell pellets were resuspended in 100 uL of PBS after centrifugation. The cell suspensions were treated with a cocktail of monoclonal antibodies, including 5 uL of CD14-*Phycoerythrin–cyanine 7* (PC7; Product No: A22331, Brea, CA, USA), 5 uL CD16-*Phycoerythrin-Texas Red-X* (ECD; Product No: B49216, Brea, CA, USA), 5 uL CD36-*Fluorescein isothiocyanate* (FITC; Product No: B49201, Brea, CA, USA), 2,5 uL CD163-*Phycoerythrin* (PE; Product No: 326506, Biolegend, CA, USA), 2,5 uL CD206-*Allophycocyanin* (APC; Product No: 321110, Biolegend, CA, USA), and 1 uL of Fixable Viability Dye *eFluor™ 780* (Product No: 65-0865-18; Thermo Fisher, Waltham, MA, USA) for 30 min, with analysis being performed within 2 h of staining. The concentrations of antibodies were optimized during previous research in our laboratory. The flow cytometric analyses were performed on a Navios Ex3/10 Beckman Coulter flow cytometer (Beckman Coulter, Brea, CA, USA). The mean fluorescence intensity (MFI) for macrophage surface marker expression was then quantified using FlowJo™ v10 Software (BD Life Sciences). 13 samples were used for flow cytometry.

### Gene expression – quantitative real-time PCR (qRT-PCR)

Cells for qRT-PCR were obtained as described above. RNA was isolated from harvested cells with TriReagent (Molecular Research Center, Cincinnati, OH, USA), and the concentration of RNA was quantified using the NanoDrop 2000 spectrophotometer (Thermo Scientific). Before reverse transcription, RNA was treated with DNase I (Sigma Aldrich, St. Louis, MO, USA) to avoid DNA contamination. cDNA was generated according to the manufacturer’s instructions using the HighCapacity RNA-to-cDNA Master Mix kit (Life Technologies, Carlsbad, CA, USA). Then, equal concentrations of cDNA for each PCR reaction (100 ng/ per 20 uL qRT-PCR reaction mix) were used to quantify gene expression levels using the HOT FIREPol® EvaGreen® qPCR Mix Plus (ROX) (Solis BioDyne, Tartu, Estonia), determined with the Corbett Life Science Rotor Gene 3000 (Qiagen, Venlo, The Netherlands). Quantitative RT-PCR analyses were conducted in triplicate from each sample. The expression of classical pro-inflammatory genes (NFκB, IL-1β, IL-6, iNOS, TNFα, and MCP-1) and anti-inflammatory Arg-1 and TGFβ were quantified and normalized by Beta-2-microglobulin (b2m) expression levels. All primers were synthesized in GENERI BIOTECH (Hradec Kralove, Czech Republic) for gene sequences, see Table 1. Relative gene expression was calculated using the ΔΔCt method^25^ compared with appropriate respective unstimulated controls.

**Table 1:** The nucleotide sequence of the genes tested in this study.

Primer	Sequence (5′- 3′)
b2m (F) b2m (R)	TCTCTCTTTCTGGCCTGGAG AATGTCGGATGGATGAAACC
NF-κB (F) NF-κB (R)	ATGGCTTCTATGAGGCTGAG GTTGTTGTTGGTCTGGATGC
IL-1β (F) IL-1β (R)	ACAGATGAAGTGCTCCTTCCA GTCGGSGSTTCGTSGCTGGAT
IL-6 (F) IL-6 (R)	AGCAGCAAAGAGGCACTGGCA TGAGGAACAAGCCAGAGCTGTGC
TNFα (F) TNFα (R)	TCTTCTCGAACCCCGAGTGA CCTCTGATGGCACCACCAG
NOS2 (F) NOS2 (R)	CAARGGCAACATCAGGTCGG TGAAGGATTCTGCAGCCGAG
MCP-1 (F) MCP-1 (R)	AGAAGCTGTGATCTTCAAGACC AGCTGCAGATTCTTGGGTTG
Arg-1 (F) Arg-1 (R)	ACAGTTGGCAATTGGAAGCA CACCCAGATGACTCCAAGATCAG
TGF-β (F) TGF-β (R)	TCGCCAGAGTGGTTATCTTTT TAGTGAACCCGTTGATGTCC

### Cytokine quantification – ELISA tests

ELISA MAX™ Deluxe Set (Biolegend) kits were used to quantify macrophages’ production of cytokines (IL-1β, IL-6, TNFα, and IL-10). Samples were processed according to the instructions in the attached protocols. ELISA assays were conducted in duplicates from each sample. Absorbance was measured on a microplate reader (Multi-DetectionMicroplate Reader, Synergy 2, BioTek, Bad Friedrichshall, Germany) at 450 nm and 570 nm wavelengths.

### Quantification of inducible nitric oxide synthase (iNOS) activity – Griess assay

The activity of iNOS was assessed with the Griess assay^24^. The Griess assay detects nitrite ions formed as a byproduct of nitric oxide, reflecting the enzyme’s activity (iNOS). The media were incubated with nitrate reductase from the mold *Aspergillus Niger* (50 mU/mL, Sigma-Aldrich, St. Louis, MO, USA) and NADPH (Sigma-Aldrich, St. Louis, MO, USA, 100 µmol/L) for 30 min at room temperature. After reduction, the samples were incubated with methanol and diethyl ether (a 3:1 mixture, v/v) for 1 h at 4 °C. After incubation, samples were centrifuged (10 000 g, 10 min at 4 °C), and the supernatants were used for nitrite determination. A Griess reagent; a mixture of solution A (0.1% *N*-1-naphthyl ethylenediamine dihydrochloride in water) and solution B (1% sulfanilamide in 5% H_3_PO_4_, 1:1, v/v) was added to the prepared samples. Griess assays were conducted in duplicate from each sample. Absorbance was measured at 540 nm using a microplate reader (Multi-DetectionMicroplate Reader, Synergy 2, BioTek, Bad Friedrichshall, Germany). A standard curve was generated with sodium nitrite at 2.5 to 150 µmol/L concentrations.

### Statistics

A two-way ANOVA test with Tukey’s multiple comparison post-hoc test was used to analyze compared groups statistically. GraphPad Prism 8 software (GraphPad Software Inc., San Diego, CA, USA) was used for the statistical analysis. The results are presented as mean ± SEM, expressed in MFI, pg/mL. µmol/L or fold change for gene expression, depending on the parameter measured. Relative gene expression was calculated using the ΔΔCt method compared with appropriate respective unstimulated controls. A confidence interval (CI) of 95% was set, and values were considered significant at *p* < 0.05. An overview of the source of variation is provided at the end of the section.

## Results

### Gene expression

To prove fluvastatin’s anti-inflammatory properties on the gene level, the gene expression of pro-inflammatory (NFκB, IL-1β, TNFα, IL-6, iNOS, and MCP-1) and anti-inflammatory (Arg-1 and TGFβ) markers was quantified.

**Fig. 1:**
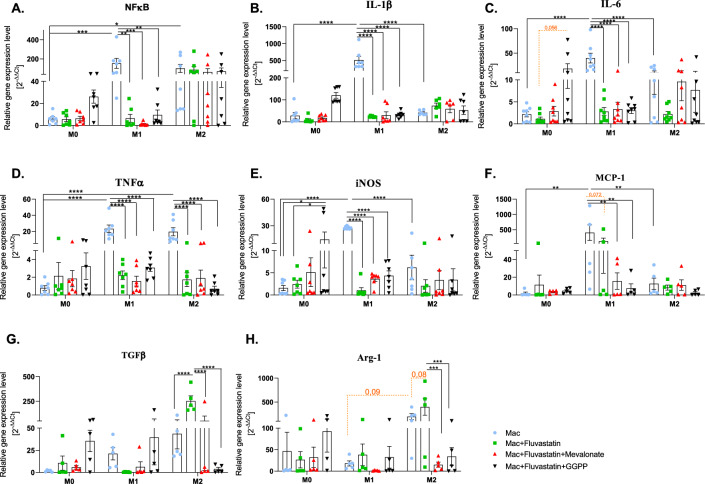
The expression of pro-inflammatory NFκB, IL-1β, IL-6, TNFα, iNOS, and MCP-1 and anti-inflammatory TGFβ and Arg-1. The effect of statin treatment on the pro-inflammatory (**A**) NFκB, (**B**) IL-1β, (**C**) IL-6, (**D**) TNFα, (**E**) iNOS, (**F**) MCP-1 and anti-inflammatory (**G**) TGFβ, (**H**) Arg-1 gene expression by macrophages. Non-stimulated macrophages (M0), M0 + statin, M0 + statin + mevalonate, M0 + statin + geranylgeranyl pyrophosphate, 48 h therapy; LPS + IFNγ stimulated macrophages (M1), M1 + statin, M1 + statin + mevalonate, M1 + statin + statin + geranylgeranyl pyrophosphate, 48 h therapy; IL-4 + IL-13 stimulated macrophages (M2), M2 + statin, M2 + statin + mevalonate, M2 + statin + geranylgeranyl pyrophosphate, 48 h treatment. The analyses were conducted in triplicate from each sample, n = 5–8. Data are presented as relative gene expression mean ± SEM. Black lines for two-way ANOVA indicate statistical significance with Tukey’s multiple comparisons post-hoc test. Orange Dotted lines indicate borderline significance. The (CI) set at 95% with exact *p*-values are provided in the text.

Focusing on gene expression of pro-inflammatory markers (NFκB, IL-1β, IL-6, iNOS, and MCP-1) (Fig. 1A–F), the results show their significant upregulation in M1 macrophages compared with M0 and M2, confirming their proper stimulation toward a pro-inflammatory state. Interestingly, TNFα expression in M2 macrophages was comparable to that of M1. Except for MCP-1, where the expression was reduced on the border of significance (Fig. 1c, *p* = 0.072), fluvastatin treatment significantly downregulated pro-inflammatory genes (NFκB, IL-1β, IL-6, iNOS, and TNFα) suggesting a potential anti-inflammatory effect. M2 macrophages maintained low expression of these markers, consistent with their anti-inflammatory function.

In particular, NFκB was the most highly expressed in M1 macrophages (Fig. 1A), reaching statistical significance compared with M0 macrophages (***p = 0.0005) and M2 macrophages (*p < 0.05). Fluvastatin significantly reduced NFκB expression in M1 macrophages (**p = 0.0011). The effect was not restored with either mevalonate pathway intermediate. Gene expression of IL-1β was significantly elevated in M1 compared with M0 and M2 macrophages (****p < 0.0001) and was strongly inhibited by fluvastatin in M1 macrophages (****p < 0.0001, Fig. 1B). Mevalonate and GGPP did not fully restore the expression. Similarly, IL-6 was upregulated in M1 macrophages compared to other populations (****p < 0.0001, Fig. 1C), with fluvastatin reducing IL-6 gene expression in M1 (****p < 0.0001). Although the tendency of fluvastatin to reduce the expression of IL-6 in M2 macrophages is equal to that in M1 macrophages, the data did not reach statistically significant values. The intermediates had a significant effect neither on M1 nor M2 macrophages. However, GGPP almost significantly affected the expression of IL-6 by fluvastatin-treated- M0 macrophages. Compared with M0 macrophages, TNFα gene expression was upregulated in M1 and M2 (***p < 0.0001) macrophages, but fluvastatin effectively downregulated it in both (****p < 0.0001). iNOS expression was significantly reduced by fluvastatin in M1 macrophages (***p < 0.0001). Interestingly, in the M0 macrophage subpopulation, GGPP significantly upregulated the expression of iNOS compared to M0 macrophages (*p = 0.015) and fluvastatin-treated M0 macrophages (*p = 0.03). Finally, MCP-1 was strongly expressed in M1 macrophages compared to M0 and M2 macrophages (*p = 0.021; **p = 0.0028 respectively), with fluvastatin almost significantly reducing the expression by M1 macrophages (*p* = 0.072; Fig. 1F).

When focusing on the expression of anti-inflammatory markers, fluvastatin treatment upregulated gene expression of the analyzed anti-inflammatory markers in M2 macrophages (TGFβ and Arg-1, Fig. 1G,H**).** Although the relative gene expression of both TGFβ and Arg-1 was notably higher in M2 macrophages compared with M1 and M0 macrophages, statistical significance was not reached. Treatment with fluvastatin enhanced upregulation of anti-inflammatory gene expression in M2 macrophages (TGFβ ****p < 0.0001; Arg-1 *p* = 0.08), further reinforcing their anti-inflammatory phenotype. Interestingly, both intermediates, mevalonate and GGPP, restored the effect of fluvastatin treatment on TGFβ (mevalonate ****p < 0.0001; GGPP ****p < 0.0001) and Arg-1 (mevalonate ***p = 0.0002; GGPP ***p = 0.0004) gene expression by M2 macrophages.

To further understand the effect of both variables (polarization and treatment) and their interaction on gene expression, we extended our data to analyze the source of variation. As shown in Table 2, polarization of macrophages had a significant effect on the expression of most genes, except MCP-1. Fluvastatin treatment significantly affected most of the gene expression, except IL-6. Notable, the analysis revealed the significant interaction effects on all gene expressions except MCP-1.

**Table 2:** Source of variation in gene expression analysis.

	Polarization	Treatment	Interaction
NFκB	*p = 0.0195	**p = 0.0018	*p = 0.0333
IL-1β	****p < 0.0001	****p < 0.0001	****p < 0.0001
IL-6	***p = 0.0009	(ns) 0.1508	****p < 0.0001
TNFα	****p < 0.0001	**p = 0.0015	****p < 0.0001
iNOS	***p = 0.0006	*p = 0.0379	****p < 0.0001
MCP-1	(ns) 0.1461	*p = 0.0322	(ns) 0.113
TGFβ	**p = 0.0021	****p < 0.0001	****p < 0.0001
Arg-1	*p = 0.0395	**p = 0.0039	**p = 0.0097

The data in the table represent how the two factors (polarization and treatment) affect the gene expression level. The interaction between the two variables describes the treatment‘s dependency on the polarization state of macrophages. The results were analyzed using a two-way ANOVA test.

### Cytokine production

**Fig. 2:**
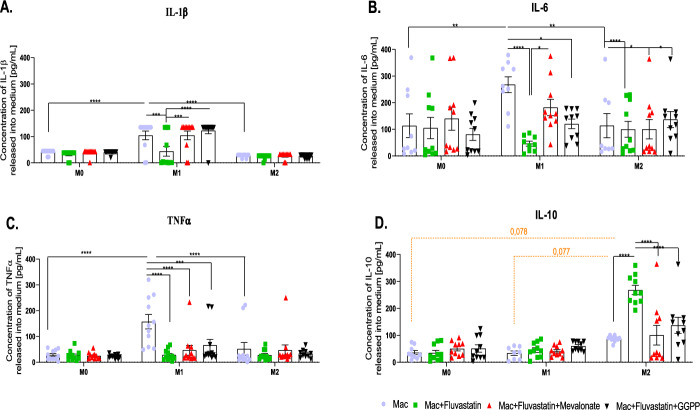
Release of pro-inflammatory IL-1β, TNFα, IL-6, and anti-inflammatory IL-10 cytokines into medium. Effect of statin treatment on the release of pro-inflammatory (**A**) IL-1β, (**B**) TNFα, (**C**) IL-6 and anti-inflammatory (**D**) IL-10 cytokines by macrophages with or without statin into the medium. Non-stimulated macrophages (M0), M0 + statin, M0 + statin + mevalonate, M0 + statin + geranylgeranyl pyrophosphate, 48 h therapy; LPS + IFNγ stimulated macrophages (M1), M1 + statin, M1 + statin + mevalonate, M1 + statin + statin + geranylgeranyl pyrophosphate, 48 h treatment; IL-4 + IL-13 stimulated macrophages (M2), M2 + statin, M2 + statin + mevalonate, M2 + statin + geranylgeranyl pyrophosphate, 48 h treatment. ELISA assays were conducted in duplicates from each sample, n = 10–11. Data are presented as mean ± SEM pg/mL. Black lines for two-way ANOVA indicate statistical significance with Tukey’s multiple comparisons post-hoc test. Orange Dotted lines indicate borderline significance. The (CI) set at 95% with exact *p*-values are provided in the text.

The pro-inflammatory and anti-inflammatory cytokines (IL-1β, IL-6, TNFα, IL-10) released into the culture medium were measured via ELISA tests to verify the anti-inflammatory effect of statin treatment on the protein level. The inflammatory cytokines were strongly produced by pro-inflammatory M1 macrophages, compared with M2 and M0 macrophages (IL-1β and TNFα **** *p* < 0.0001; IL-6 **p = 0.0073 and **p = 0.0053, respectively) (Fig. 2).

Fluvastatin treatment significantly reduced the production of IL-1β by M1 macrophages, compared with untreated M1 macrophages (****p < 0.0001) (Fig. 2A). The mevalonate and GGPP restored the production of IL-1β by statin-treated M1 macrophages (***p = 0.001; **** < 0.0001, respectively) to a level similar to statin-untreated M1 macrophages. IL-6, whose production was significantly reduced by fluvastatin-treated M1 macrophages (***p < 0.001) and was restored considerably by mevalonate (*p = 0.03). The production of IL-6 by M0 and M2 was not significantly affected by fluvastatin therapy (Fig. 2B). The last pro-inflammatory cytokine to be analyzed was TNFα (Fig. 2C). Its production by M1 macrophages was significantly reduced after statin treatment (****p < 0.0001).

To further demonstrate fluvastatin’s ability to enhance anti-inflammatory responses, we investigated its effect on anti-inflammatory IL-10 production by macrophages (see Fig. 2D). Compared with M0 and M1 macrophages, M2 macrophages produced higher levels of IL-10 cytokines (*p* = 0.08), yet the significance was not reached. Statin treatment three times upregulated production of IL-10 by M2 macrophages (****p < 0.0001). The production of IL-10 by statin-treated M2 macrophages was decreased in the presence of both mevalonate and GGPP intermediates (****p < 0.0001) to levels comparable to those of fluvastatin-untreated M2 macrophages. Although fluvastatin-treated M1 macrophages tended to produce increased levels of IL-10, statistical significance was not reached.

The analysis of the source of variation unveiled the crucial dependence of all cytokines (except IL-6) production on the polarization state of macrophages (***p < 0.0001). The treatment significantly affected the production of all cytokines. The interaction between the two variables was significant in all tested markers (see Table 3).

**Table 3:** Source of variation in cytokine production analysis.

	Polarization	Treatment	Interaction
IL-1β	****p < 0.0001	**p = 0.0011	**p = 0.0027
IL-6	(ns) 0.1162	*p = 0.0247	*p = 0.0205
TNFα	****p < 0.0001	***p = 0.0006	**p = 0.0013
IL-10	****p < 0.0001	****p < 0.0001	****p < 0.0001

Data in the table represent how the two factors (polarization and treatment) affect cytokine production. The interaction between two variables describes the dependency of the treatment on the polarization state of macrophages. Analyzed by two-way ANOVA test.

### Activity of iNOS

**Fig. 3:**
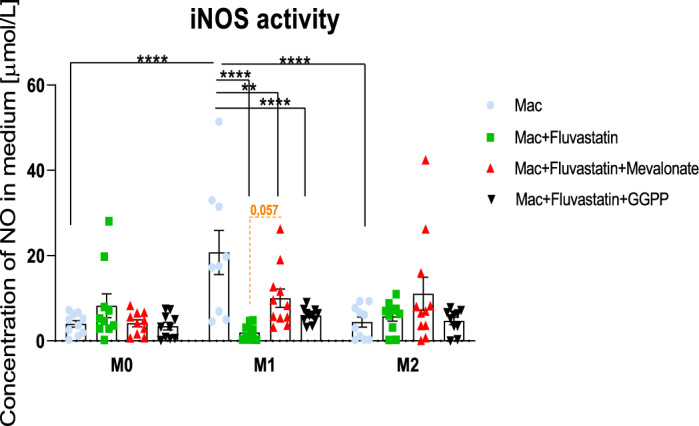
The activity of iNOS. The effect of statin treatment on the activity of inducible nitric oxide (NO) synthase quantified by the concentration of NO released by stimulated macrophages with or without statin into the medium (n = 11). Non-stimulated macrophages (M0), M0 + statin, M0 + statin + mevalonate, M0 + statin + geranylgeranyl pyrophosphate, 48 h therapy; LPS + IFNγ stimulated macrophages (M1), M1 + statin, M1 + statin + mevalonate, M1 + statin + statin + geranylgeranyl pyrophosphate, 48 h therapy; IL-4 + IL-13 stimulated macrophages (M2), M2 + statin, M2 + statin + mevalonate, M2 + statin + geranylgeranyl pyrophosphate, 48 h treatment. Griess assays were conducted in duplicates from 11 samples. Data are presented as mean ± SEM (µmol/L). Statistical significance is indicated by black lines for two-way Anova with Tukey’s multiple comparisons post-hoc test. Orange Dotted lines indicate borderline significance. The (CI) set at 95% with exact *p*-values are provided in the text.

The production of large amounts of NO is an essential marker of inflammation in macrophages. Consistent with general knowledge, pro-inflammatory M1 macrophages produced the highest amount of NO; M1 (***p < 0.0001) (Fig. 3). Statin treatment significantly reduced the concentration of NO released into the medium by M1 macrophages (****p < 0.0001). The intermediates did not significantly restore the iNOS activity, however mevalonate almost reached the border of significance (*p* = 0.056).

The analysis of the source of variation unveiled the equal dependence of iNOS activity on polarization and treatment. The interaction between the variables is strongly significant (****p < 0.0001; see Table 4).

**Table 4:** Source in variation in activity of NO synthase.

	Polarization	Treatment	Interaction
iNOS	*p = 0.0106	*p = 0.0167	****p < 0.0001

Data in the table represent how the two factors (polarization and treatment) affect the activity of inducible NO synthase. The interaction between two variables describes the dependency of the treatment on polarization state of macrophages. Analysed by two-way ANOVA test.

### Surface marker expression

**Fig. 4:**
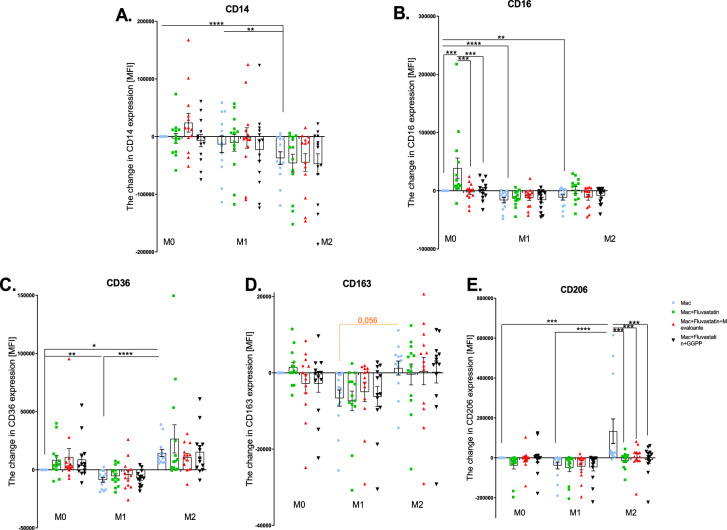
Expression of the surface markers CD14, CD16, CD36, CD163 and CD206. The effect of statin treatment on the expression of surface markers: (**A**) CD14, (**B**) CD16, (**C**) CD36, (**D**) CD163, and (**E**) CD206 by macrophages. Non-stimulated macrophages (M0), M0 + statin, M0 + statin + mevalonate, M0 + statin + geranylgeranyl pyrophosphate, 48 h therapy; LPS + IFNγ stimulated macrophages (M1), M1 + statin, M1 + statin + mevalonate, M1 + statin + statin + geranylgeranyl pyrophosphate, 48 h treatment; IL-4 + IL-13 stimulated macrophages (M2), M2 + statin, M2 + statin + mevalonate, M2 + statin + geranylgeranyl pyrophosphate, 48 h therapy. Surface markers expression analysis was conducted on 13 samples. Data are presented as MFI ± SEM. Statistical significance is indicated by black lines for two-way Anova with Tukey’s multiple comparisons post-hoc test. Orange Dotted lines indicate borderline significance. The (CI) set at 95% with exact *p*-values are provided in the text.

Finally, to test the effect of statin treatment on the expression of the surface markers (CD14, CD16, CD36, CD163, and CD206) in macrophages, mean fluorescence intensity (MFI) was analyzed. The changes in surface marker expression were quantified compared with unstimulated M0 macrophages (Fig. 4).

Compared with M0 macrophages, the intensity of CD14 expression was weaker on M1 and M2 surface (****p < 0.0001), with stronger expression on M1 macrophages, as compared with M2 macrophages (**p = 0.006). Data demonstrate that fluvastatin treatment did not have any significant effect on CD14 expression in either of the macrophage subtypes. Likewise, the intermediates did not affect the expression of CD14 on the surface of fluvastatin-treated macrophages (Fig. 4A). The intensity of CD16 was almost equal on the surface of M1 and M2 macrophages (Fig. 4B), however was significantly lower compared to M0 macrophages (****p < 0.0001 and **p = 0.0012, respectively). Fluvastatin significantly increased the expression of CD16 on M0 surface (***p < 0.0003), and this effect was almost fully restored with mevalonate (***p = 0.0001) and GGPP (***p = 0.0004). The expression of CD36 was more intensive on M2 macrophages surface compared to M1 macrophages (****p < 0.0001) and M0 (*p = 0.022) macrophages. Neither fluvastatin, nor intermediates treatment affected the expression of CD36 in any of the studied macrophage populations (Fig. 4C).

The surface marker CD163, classically considered an anti-inflammatory, expressed more strongly on the surface of M2 macrophages (Fig. 4D), compared with M1 macrophages (*p* = 0.056). Fluvastatin, nor mevalonate pathway intermediates treatment did not affect the expression of CD163 by any macrophage subpopulation. Finally, CD206, another marker classically considered anti-inflammatory, was strongly expressed on the M2 macrophage surface, compared with M1 (****p < 0.0001) and M0 (***p = 0.0004) macrophages and was reduced after fluvastatin treatment (***p = 0.0002; Fig. 4E).

While the expression of surface markers was dependent on the polarization state of macrophages in each case, the treatment affected expression only of two markers (CD16 and CD206) (Table 5). Also, the interaction between the variables was proved only in these two markers.

**Table 5:** Source in variation in surface markers expression analysis.

	Polarization	Treatment	Interaction
CD14	****p < 0.0001	(ns) 0.4736	(ns) 0.9407
CD16	****p < 0.0001	**p = 0.0016	*p = 0.0398
CD36	****p < 0.0001	(ns) 0.3188	(ns) 0.5839
CD163	***p = 0.0003	(ns) 0.9452	(ns) 0.8374
CD206	**p = 0.0075	***p = 0.0002	*p = 0.0248

Data in the table represent how the two factors (polarization and treatment) affect the expression of surface markers. The interaction between two variables describes the dependency of the treatment on polarization state of macrophages. Analysed by two-way ANOVA test.

## Discussion

Numerous studies have reported both anti-^12,25–28^ and pro-inflammatory^29–31^ effects of statins, with Fu et al. suggesting that these opposing effects may be explained by different isoprenylation mechanisms^23^.

Our study highlights the potent anti-inflammatory effects of fluvastatin, as it suppresses pro-inflammatory markers in M1 macrophages while simultaneously enhancing the expression of anti-inflammatory markers in M2 macrophages. Notably, our findings indicate that fluvastatin does not directly induce macrophage polarization in the absence of specific polarization stimuli. Instead, these results suggest that fluvastatin primarily modulates the function of already polarized macrophages rather than driving their differentiation per se.

Furthermore, our data reveal that at least some of these effects are mediated through the mevalonate pathway, as supplementation with pathway intermediates partially restored fluvastatin-induced changes, primarily at the protein level rather than at the gene expression level. Importantly, variance analysis demonstrated a strong interaction between polarization state and fluvastatin treatment across all analyses, with the exception of flow cytometry, which assessed surface marker expression.

To the best of our knowledge, this is the first study demonstrating that the immunomodulatory effects of fluvastatin are contingent on the pre-existing polarization state of macrophages, rather than directly inducing their polarization. Moreover, we believe this is among the first studies to specifically examine the immunomodulatory properties of fluvastatin in M2 macrophages, which are typically considered anti-inflammatory. These findings underscore the novelty of our study and its contribution to the current understanding of fluvastatin’s role in macrophage regulation.

A key finding of our study is the significant reduction in pro-inflammatory genes (NFκB, IL-1β, IL-6, TNFα and iNOS expression by M1 macrophages after fluvastatin treatment, MCP-1 expression was also reduced, though this reduction was borderline significant (*p* = 0.072). NFκB is a well-established mediator of inflammatory responses, and its downregulation by statins aligns with previous reports that demonstrate the ability of statins to inhibit NFκB signaling in various cell types^26,32–34^. The gene expression of the pro-inflammatory markers IL-1β, IL-6, iNOS and TNFα was also significantly downregulated by fluvastatin, particularly in M1 macrophages. This supports the idea that statins have broad anti-inflammatory effects^23,26,32,35^. Importantly, the gene expression of classical anti-inflammatory markers, TGFβ and Arg-1, by M2 macrophages was increased following fluvastatin treatment, further supporting the idea of anti-inflammatory properties of statins, since the increased expression of Arg-1 by macrophages following statin treatment was documented also in animal models^36^. Based on their results, the authors suggest the ability of statins to shift M1 macrophages toward the M2 phenotype. However, our results do not support this conclusion, as fluvastatin did not increase the gene (Fig. 1) and surface markers (Fig. 4) expression or production (Fig. 2) of anti-inflammatory markers by M1 macrophages. While increased TGFβ gene expression after statin therapy was documented by other authors^37,38^, a study focusing on the effect of statin on the expression of this marker in cancer cells documented an opposite trend with statins inhibiting TGFβ expression^39^. Interestingly, we documented a similar trend in pro-inflammatory M1 macrophages (not significant). Taken together, these findings suggest the crucial importance of taking into account the type and phenotype of the cell, as well as the intrinsic factors of the studied organism.

Intermediates did not significantly affect the reduced gene expression of pro-inflammatory genes by M1 macrophages; however, they reversed the effect of fluvastatin on expression of anti-inflammatory genes (see Fig. 1G,H). These results let us conclude that the expression of anti-inflammatory genes was rather affected by an isoprenoid pathway, while pro-inflammatory genes by any other signaling pathway. The important observation was, that GGPP tended to increase the expression of most of the genes, both pro- and anti-inflammatory by M0 macrophages. This aligns with the effect of isoprenoids (thus also GGPP) on innate immune responses via affecting small GTPases^40,41^.

Our findings emphasize the critical role of macrophage polarization in determining fluvastatin’s immunomodulatory effects as show source of variation statistics. Fluvastatin modulates inflammatory and regulatory pathways in a polarization-dependent manner at the gene expression level (e.g., IL-1β, TNFα, iNOS, and TGFβ; *p* < 0.01), cytokine production, and inducible NO synthase (iNOS) activity. However, it does not significantly influence surface marker expression, except for CD16 and CD206, which were selectively altered in M0 and M2 macrophages, respectively. Interestingly, MCP-1 expression was independent of polarization and polarization-treatment interaction but was significantly affected by fluvastatin (*p* < 0.05). This may be due to MCP-1’s broad role in monocyte recruitment, driven by both pro- and anti-inflammatory cytokines^42^.

The selective regulation of CD16 and CD206 suggests distinct underlying mechanisms. CD16 in non-polarized macrophages may be particularly sensitive to changes in lipid composition and protein prenylation, whereas CD206 in M2 macrophages may rely more on isoprenoid synthesis and downstream signaling. This highlights the need for further research into intracellular transport and receptor incorporation into the membrane.

At the protein level (Fig. 2), our data reliably demonstrate the strong effect of fluvastatin treatment on reduced production of the pro-inflammatory cytokines IL-1β, IL-6, and TNFα M1 macrophages while enhancing the production of the anti-inflammatory cytokine IL-10 by M2 macrophages. These findings are consistent with Fu et al.^23^, however extended for three different subsets of macrophages. Although intermediates failed to restore pro-inflammatory gene expression, they successfully reversed the suppression of IL-1β and IL-6 protein production. The differential effect of intermediates on gene expression and cytokines production further supports the idea that fluvastatin affects inflammatory responses through multiple mechanisms. These findings suggest that post-transcriptional or translational mechanisms, such as intracellular transport or secretion pathways, may play a crucial role in cytokine regulation. It reinforces the need to investigate how fluvastatin affects intracellular receptor transport and membrane incorporation, as these processes could explain the observed inconsistencies between gene expression and cytokine production.

We also focused on the impact of fluvastatin treatment on iNOS activity as an important proinflammatory macrophage property. It mirrors our previous results^12^, as fluvastatin significantly decreased NO production by M1 macrophages (Fig. 3). It is consistent with its ability to reduce the gene expression of iNOS by M1 macrophages (Fig. 1). Though the intermediates tended to restore the activity of iNOS, the effect was insignificant. Our data suggest that these effects partially depend on the mevalonate pathway. Our results are in contradiction with the study by Ikeda et al.^43^, where statins increased iNOS activity in cardiac myocytes in vitro. However, the role of iNOS in these myocytes differs from that in macrophages^44^ thus the mechanism of statin action also differs.

The limited impact of fluvastatin on macrophage surface marker expression suggests that its immunomodulatory effects may not primarily operate through direct regulation of surface marker genes. However, the significant reduction in CD206 expression on M2 macrophages is intriguing, especially since it contradicts previous findings^56^. The fact that mevalonate pathway intermediates did not reverse this effect implies that CD206 downregulation is not solely dependent on isoprenoid depletion but may involve alternative mechanisms. One possible explanation is that fluvastatin primarily affects intracellular trafficking and receptor recycling rather than directly regulating transcription. CD206 undergoes constant internalization and recycling^45^, and statins might interfere with these processes by altering lipid raft composition, vesicular transport, or endosomal sorting.

The unexpected increase in CD16 expression in M0 macrophages after fluvastatin treatment and its near-complete reversal by both mevalonate and GGPP suggests that this effect is due to disruptions in the mevalonate pathway—particularly the depletion of GGPP, which plays a key role in cell signaling and receptor regulation^41,46^. The question of why the CD16 expression was adjusted only on M0 macrophages might be explained as follows: Compared to M0, polarized macrophages have already been activated on the surface marker expression level. This might make them less sensitive to fluvastatin-induced changes; M0 macrophages might rely more on isoprenoid-mediated signaling; the lack of polarization in M0 macrophages might leave CD16 more susceptible to adjustments by fluvastatin treatment.

The (co)-incubation time and concentration were adopted from Fu et al.^24^ and tested during the preliminary experiments (data not shown) for the publication published by Kauerova et al. earlier^8^. The preliminary data, however, clearly demonstrated that simultaneous incubation with polarizing stimuli leads to stronger immunomodulatory effects. The co-incubation more accurately reflects the in vivo scenario where statins are present alongside cytokines and other immune mediators.

This study has several limitations. While it investigates the immunomodulatory effects of fluvastatin in human macrophages differentiated from blood monocytes using well-established polarization protocol, it only examines the effects of statins on specifically polarized macrophage subtypes, which may not fully represent the complexity of macrophage phenotypes in vivo. One limitation of this study is the exclusive use of fluvastatin. Although fluvastatin is prescribed less frequently^47,48^, it was selected for its advantageous properties, including water solubility (eliminating the need for organic solvents), increased lipophilicity (enhancing cellular uptake), and chemical stability in culture^3,49,50^. Moreover, it is generally well tolerated and an alternative for patients intolerant to more commonly used statins. Also, fluvastatin shows a lower risk of type 2 diabetes compared to other statins (Fluvastatin also indicates a lower risk of development of type 2 diabetes compared to other statins^6^ . Another limitation is using a single incubation time and concentration, which may not capture the full spectrum of fluvastatin’s effects. The experimental design was based on Fu et al.^23^, and preliminary findings by Kauerova et al.^12^, demonstrating that co-incubation with polarizing stimuli better reflects in vivo conditions, where statins interact with cytokines and immune mediators. While the direct clinical relevance of the chosen dose and incubation period remains uncertain, the shared mechanism of action among statins suggests that these findings may extend beyond fluvastatin.

## Conclusion

This study investigates the immunomodulatory effects of fluvastatin on macrophages, providing a novel perspective by examining its impact on fully polarized pro-inflammatory M1, fully polarized anti-inflammatory M2, and non-polarized M0 peripheral blood monocyte-derived macrophages. Fluvastatin exerts a potent anti-inflammatory effect on both M1 and M2 macrophages, reducing the pro-inflammatory properties of M1 while enhancing the anti-inflammatory properties of M2 without inducing a phenotypic switch between polarization states. Its effects are evident at the gene expression and protein levels, influencing cytokine production and iNOS activity, though its impact on surface marker expression remains inconsistent. The immunomodulatory properties of fluvastatin appear to be dependent on macrophage polarization status, and it does not independently drive polarization without specific polarizing stimuli. Notably, the lack of consistent effects on surface marker expression and the inconsistent restoration by mevalonate pathway intermediates suggest further research into its influence on intracellular transport and receptor incorporation into the membrane. Additionally, investigating alternative signaling pathways beyond the mevalonate pathway could enhance understanding of its immunomodulatory mechanisms. While these findings might be likely extended to other statins due to their shared mechanism of action, future studies should explore the effects of more commonly used statins to confirm their broader relevance.

## Data Availability

The data are available from the corresponding author on reasonable request.
